# Supplementary material for: The interactome of intact mitochondria by cross-linking mass spectrometry provides evidence for coexisting respiratory supercomplexes
Source: Mol Cell Proteomics. 2017 Dec 8;17(2):216–32. doi: 10.1074/mcp.RA117.000470 (PMC5795388; doi:10.1074/mcp.RA117.000470)
Supplement: Supplemental Data [file supp_RA117.000470_133922_0_supp_23978_fzffwf.zip › spectra_annotation/mito_DR_spectra_annotation/406-2-3-1-4-1.pdf]

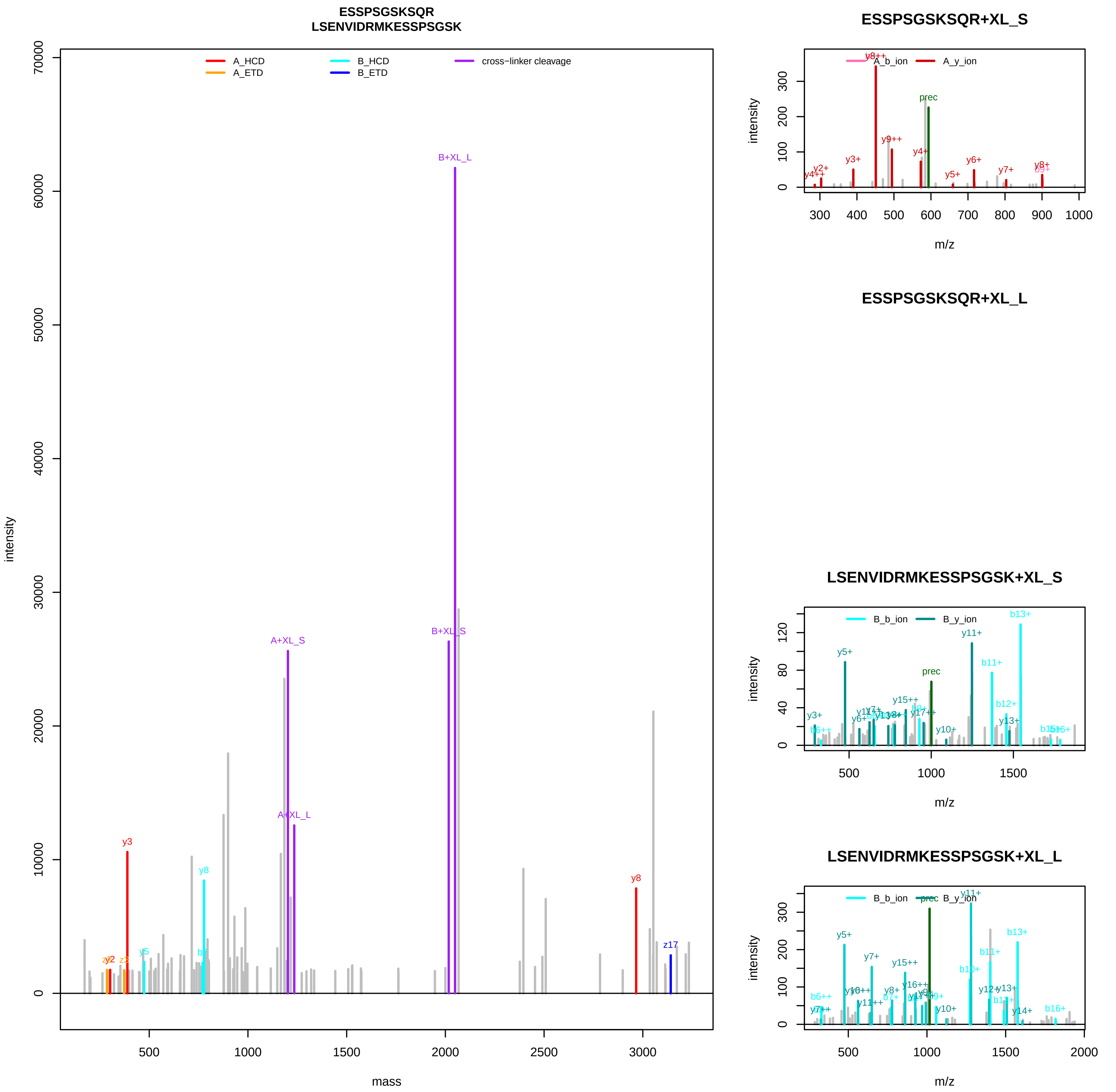
ESSPSGSKSQR
LSENVIDRMKESSPSGSK
A_HCD
A_ETD
B_HCD
B_ETD
cross−linker cleavage
B+XL_L
B+XL_S
A+XL_S
A+XL_L
y3
y8
z17
intensity
mass
ESSPSGSKSQR+XL_S
A_b_ion
A_y_ion
prec
y8++
y9++
y3+
y4+
y6+
y5+
y7+
y8+
m/z
ESSPSGSKSQR+XL_L
LSENVIDRMKESSPSGSK+XL_S
B_b_ion
B_y_ion
b13+
y11+
b11+
y5+
b12+
y13+
y15++
y3+
y10+
LSENVIDRMKESSPSGSK+XL_L
y7+
y15++
b10+
y12+
y14+
b16+
